# Binding of Plasminogen to *Streptococcus suis* Protein Endopeptidase O Facilitates Evasion of Innate Immunity in *Streptococcus suis*

**DOI:** 10.3389/fmicb.2021.694103

**Published:** 2021-07-08

**Authors:** Yang Zhou, Kang Yan, Chengfeng Sun, Feng Liu, Wei Peng, Huanchun Chen, Fangyan Yuan, Weicheng Bei, Jinquan Li

**Affiliations:** ^1^State Key Laboratory of Agricultural Microbiology, College of Veterinary Medicine, Huazhong Agricultural University, Wuhan, China; ^2^College of Fisheries, Huazhong Agricultural University, Wuhan, China; ^3^The Cooperative Innovation Center for Sustainable Pig Production, Wuhan, China; ^4^Key Laboratory of Prevention and Control Agents for Animal Bacteriosis, Ministry of Agriculture, Institute of Animal Husbandry and Veterinary Sciences, Hubei Academy of Agricultural Sciences, Wuhan, China

**Keywords:** *Streptococcus suis*, plasminogen, SsPepO, C3b, ϵ-ACA, immune evasion

## Abstract

The Gram-positive bacterial species *Streptococcus suis* is an important porcine and human pathogen that causes severe life-threatening diseases associated with high mortality rates. However, the mechanisms by which *S. suis* evades host innate immunity remain elusive, so identifying novel virulence factors involved in immune evasion is crucial to gain control over this threatening pathogen. Our previous work has shown that *S. suis* protein endopeptidase O (SsPepO) is a novel fibronectin-binding protein. Here, we identified that recombinant SsPepO binds human plasminogen in a dose-dependent manner. Moreover, the binding of SsPepO and plasminogen, upon the activation of urokinase-type plasminogen activator, generated plasmin, which could cleave complement C3b, thus playing an important role in complement control. Additionally, a *SspepO*-deficient mutant showed impaired adherence to plasminogen as well as impaired adherence to and invasion of rat brain microvascular endothelial cells compared with the wildtype strain. We further found that the *SspepO*-deficient mutant was efficiently killed by human serum and blood. We also confirmed that the *SspepO*-deficient mutant had a lower mortality rate than the wildtype strain in a mouse model. In conclusion, these results indicate that SsPepO is a novel plasminogen-binding protein that contributes to *S. suis* immune evasion.

## Introduction

*Streptococcus suis* is an important emerging zoonotic pathogen in swine and humans that causes meningitis, endocarditis, arthritis, pneumonia, bacteremia, and sudden death (Segura, 2009). In 2005, a large outbreak (215 cases) of human *S. suis* infections occurred in Sichuan Province, China (Yu et al., 2006; Chen et al., 2007; Segura, 2009). Seven years earlier, in 1998, an outbreak of human *S. suis* infections was reported in Jiangsu Province, China (Normile, 2005; Feng et al., 2010). *S. suis* has also caused human cases of infection in Vietnam, Thailand, North America, United Kingdom, Australia, and New Zealand (Wisselink et al., 2000; Wertheim et al., 2009; Gottschalk et al., 2010; Hoa et al., 2013; Hatrongjit et al., 2015; Dong et al., 2021). To cause meningitis, *S. suis* needs to disseminate into the bloodstream and overcome host immune defenses. However, the exact mechanism by which *S. suis* survives in the host blood and evades host innate immune defenses is still unclear.

Extracellular matrix (ECM) is a composite of the secreted products of resident cells found in every tissue and organ (Westerlund and Korhonen, 1993). Like other Gram-positive bacteria, *S. suis* can express adhesion proteins capable of binding to various components of the host ECM to mediate adherence to host cells. The ECM-binding proteins of bacteria recognize ECM, allowing bacterial adhesion to host cells in tissue, and these proteins generally make important contributions to the infection as well as to host–pathogen interactions (Westerlund and Korhonen, 1993). For example, *S. suis* muramidase-released protein (MRP) is a multi-functional fibrinogen-binding protein and an important virulence factor of *S. suis* that promotes the development of *S. suis* meningitis and enhances the survival of *S. suis* in host blood (Wang et al., 2015; Pian et al., 2016). During adhesion, ECM proteins, such as fibronectin, usually serve as mediators between host cells and *S. suis* (Jobin et al., 2004; Musyoki et al., 2016). Furthermore, a number of *S. suis* proteins that can interact with fibronectin or plasminogen have been shown to be important virulence factors of *S. suis*, a strategy that is commonly employed by many pathogens (Gottschalk and Segura, 2000; Musyoki et al., 2016). These proteins include *S. suis* adherence and virulence factors, enolase (Esgleas et al., 2008; Sun et al., 2016), GAPDH (Brassard et al., 2004; Jobin et al., 2004), and Ssa (Li et al., 2013). Therefore, studying the fibronectin- and plasminogen-binding proteins of *S. suis* that interact with host cells may advance understanding of the pathogenesis of *S. suis*-induced infection.

The 92-kDa glycoprotein plasminogen circulates as an inactive proenzyme in human serum (Singh et al., 2015; Peetermans et al., 2016). Plasminogen is an important component of the fibrinolytic system and contributes to fibrinolysis, homeostasis, and ECM degradation (Agarwal et al., 2013). Furthermore, plasminogen activation into plasmin by plasmin activators [e.g., urokinase plasmin activator (uPA)] also inhibits complement, efficiently cleaving C3 and C5 (Lahteenmaki et al., 2001; Barthel et al., 2012a). Many pathogenic microbes utilize the protease activity of plasminogen for immune evasion (Singh et al., 2012, 2013).

Our previous studies have shown that *S. suis* protein endopeptidase O (SsPepO) contributes to the development of *S. suis* meningitis by promoting fibronectin-mediated binding to and penetration of the blood–brain barrier (Liu et al., 2017). In this study, we identified SsPepO as a novel plasminogen-binding protein. We also found that fibronectin and plasminogen did not compete with each other for binding SsPepO. Moreover, upon activation of SsPepO-bound plasminogen by uPA, the plasmin that was generated subsequently cleaved complement C3b, thus enhancing the survival of serum- and blood-susceptible *S. suis*. In conclusion, these results show that SsPepO, as a plasminogen-binding protein, plays a role in aiding in the immune evasion of *S. suis*.

## Materials and Methods

### Bacterial Strain Culture Conditions, Primers, Plasmids, and Cell Lines

All strains and plasmids used in the present study are described in Table 1. *S. suis* 2 was grown at 37°C in either tryptic soy broth or agar (TSA; Difco Laboratories, Franklin Lakes, NJ, United States) supplemented with 10% (vol/vol) newborn bovine serum and 50 μg/ml spectinomycin. *Escherichia coli* DH5α (TaKaRa, Beijing, China) was grown in Luria-Bertani medium supplemented with 100 μg/ml spectinomycin or 50 μg/ml ampicillin.

**TABLE 1:** Bacterial strains, plasmids, and antibody used in this study.

Strain or plasmid	Relevant characteristics^*a*^	Source
**Strains**		
***S. suis***		
SC19	Virulent strain isolated from the brain of a dead pig; Serotype 2	Laboratory collection
Δ*SsPepO*	The *SsPepO* deletion mutant of SC19	Laboratory
CΔ*SsPepO*	Complemented strain of Δ*SsPepO*; Spc^*R*^	Laboratory
***E. coli***		
DH5α	Cloning host for recombinant vector	TaKaRa
BL21 (DE3)	The expression host for pET-30a and their derivative	TransGen
**Plasmids**		
Pet-30a	Expression vector; Kan^*R*^	Novagen
Pet30a-*SsPepO*	pET30a containing the *SsPepO* locus	Laboratory
**Antibody**		
Anti-SsPepO	Anti-SsPepO ployclonal rabit antibody	Laboratory

*^*a*^Restriction sites are underlined; Kan^*R*^, Kanamycin resistance; Spc^*R*^, spectinomycin resistance.*

### Expression and Purification of Recombinant Protein

The expression and purification of recombinant SsPepO protein were performed as described previously (Liu et al., 2017). The native protein was expressed by the addition of 1 mM IPTG to exponential-phase cultures at 37°C for 4 h. After sonication and centrifugation, the expressed protein was purified by Ni-NTA affinity chromatography (GE Healthcare, Medical City North Hills, TX, United States). The quality of purified SsPepO protein was determined by sodium dodecyl sulfate–polyacrylamide gel electrophoresis (SDS-PAGE), and SsPepO was then stored at −80°C until use.

### Direct Binding Assays

Direct binding assays were performed as previously described (Agarwal et al., 2013). Microtiter wells were incubated with 5 μg/ml of SsPepO, plasminogen (Hematologic Technologies, Pinewood Plaza, VT, United States), fibronectin (Hematologic Technologies, Pinewood Plaza, VT, United States), or casein (Biosharp, Shenzen, China) at 4°C overnight, and casein was used as a control. After blocking for 2 h at 37°C, SsPepO was incubated with the immobilized plasminogen or fibronectin for SsPepO binding, or plasminogen or fibronectin was incubated with the immobilized SsPepO for plasminogen or fibronectin binding. The proteins in the wells were detected with anti-SsPepO antibody, anti-plasminogen antibody (Proteintech, Wuhan, China), or anti-fibronectin antibody (Proteintech, Wuhan, China), followed by an appropriate secondary antibody (Proteintech, Wuhan, China). To investigate the effects of ionic strength and the effects of lysine analog ϵ-ACA (Sigma, Darmstadt, Germany) on plasminogen–SsPepO interactions, NaCl or ϵ-ACA, respectively, was added to the plates.

### Binding From Serum

Binding from serum assays were performed as previously described (Agarwal et al., 2013). Microtiter plates were coated overnight with SsPepO (0.25 μg/well) at 4°C. Casein was used as a control. After blocking for 2 h at 37°C, human serum was added to the wells for 1.5 h at 37°C. The samples were measured with rabbit anti-plasminogen antibody (Proteintech, Wuhan, China) followed by peroxidase-conjugated secondary antibody (Proteintech, Wuhan, China). The absorbance was measured at 490 nm.

### Plasminogen Activation and C3b Protein Degradation Assays

Plasminogen activation and C3b protein degradation were each assayed as described previously (Castiblanco-Valencia et al., 2016). For the plasminogen activation assay, a SsPepO-coated plate (5 μg/well) was incubated with plasminogen (1 μg/well) for 1.5 h at 37°C. After the wells were washed, 10 units/well of uPA (Sigma, St. Louis, MO, United States) were added, and the samples were incubated at 37°C. The activity of the newly generated plasmin was measured by using the chromogenic substrate D-Val-Leu-Lys-p-nitroanilide dihydrochloride (S-2251) (Chromogenix, Milan, Italy). The absorbance of each well in the plate was measured at OD405.

For the C3b protein degradation assay, microtiter plates were coated with SsPepO (5 μg/well) overnight at 4°C and blocked by a blocking solution for 2 h at 37°C. Plasminogen (5 μg/well) was incubated with the immobilized SsPepO for 1.5 h at 37°C. After washing, 125I-labeleld C3b together with uPA (10 units/well) were added and incubated at 37°C.

### *Streptococcus suis* Binding to Plasminogen

Strains binding to immobilized plasminogen were performed as described previously (Agarwal et al., 2013). Microtiter plates were incubated overnight with strains [10^7^ colony-forming units (CFU)] at 37°C. After being blocked for 2 h, plasminogen was added to wells for 1.5 h. The samples were measured with rabbit anti-plasminogen antibody (Proteintech, Wuhan, China) followed by peroxidase-conjugated secondary antibody (Proteintech, Wuhan, China). The absorbance was measured at 490 nm.

### Western Blotting

Whole cell extracts and culture supernatant proteins were prepared for western blotting as follows. Bacteria were grown to an OD600 of 0.8, after which the cells were pelleted by centrifugation at 5,000 × *g* for 10 min at 4°C. Whole cell pellets were suspended in 1 × SDS-PAGE sample loading buffer. After being filtered through 0.22-μm membranes, the culture supernatants were precipitated in 10% trichloroacetic acid (TCA) overnight at 4°C. All supernatant samples were centrifuged at 12,000 × *g* for 30 min at 4°C, washed with ice-cold acetone, and resuspended in 1 ml of phosphate-buffered saline (PBS). Bovine serum albumin (BSA) (150 μg/ml) was added to the culture supernatant prior to TCA precipitation as a control. Whole cell extracts and culture supernatant proteins were resolved by SDS-PAGE and transferred to nitrocellulose membranes for immunoblotting. The immobilized proteins were incubated with rabbit anti-SsPepO antibody, followed by incubation with horseradish peroxidase (HRP)-conjugated goat anti-rabbit (Cell Signaling, Danvers, MA, United States). Following stripping, the blots were re-incubated with a mouse antibody against RNA polymerase (RNAP) (Santa Cruz, Dallas, TX, United States) and mouse anti-BSA (Proteintech, Wuhan, China) and then with HRP-conjugated goat anti-mouse IgG (Cell Signaling, Danvers, MA, United States). Blots were detected with ECL (Bio-Rad, Hercules, CA, United States).

### Adherence and Invasion Assays

The bacteria were added to rat brain microvessel endothelial cell (RBMEC) monolayers at a multiplicity of infection (MOI) of 10:1 for 2 h at 37°C in 5% CO_2_. The cell monolayers were washed three times with PBS and then lysed with 0.1% saponin on ice for 20 min. The number of cell-adherent bacteria was determined by plating dilutions of the lysate on TSA plates. Fold bacterial adherence was calculated as follows: [recovered strain CFU/recovered wild-type (WT) CFU] × 100%. Invasion assay was little different from adherence assay. Briefly, the cell monolayers were incubated with penicillin G (100 units/ml) (Sigma, Darmstadt, Germany) and gentamicin (100 μg/ml) (Sigma, Darmstadt, Germany) prior to cell being lysed.

### Serum Survival Assay

Bacteria were grown to mid-log phase. Bacterial suspension (50 μl) of WT *S. suis*, Δ*SspepO*, or CΔ*SspepO* was combined with serum or heat-inactivated serum (450 μl), followed by incubation at 37°C for 1 h. After incubation, the bacteria were serially diluted and plated for quantitative determination.

### Whole Blood Bactericidal Assay

Whole blood bactericidal assays were performed as previously described with some modifications (Liu et al., 2014). Briefly, bacterial suspension of WT *S. suis*, Δ*SspepO*, or CΔ*SspepO* (100 μl, 5 × 10^5^ CFU/ml) was combined with fresh human blood (900 μl), followed by incubation at 37°C in a rotator at 10 rpm for 1 h. After incubation, the bacteria were serially diluted and plated for quantitative determination.

### Bacterial Virulence *in vivo*

Thirty female 6-week-old CD1 mice (10 mice per group) were randomly and evenly divided into three groups and injected intravenously with 8 × 10^8^ CFU of WT *S. suis*, Δ*SspepO*, or CΔSspepO. The survival time of mice was recorded for 7 days post-inoculation. All animal experiments were approved by the Laboratory Animal Monitoring Committee of Huazhong Agricultural University and performed accordingly.

### Statistical Analysis

The data were analyzed with GraphPad Prism software using one-way or two-way ANOVA analyses followed by Bonferroni’s multiple comparison test, unpaired *t*-tests followed by the Holm–*S*idak method, or by non-parametric Mann–Whitney *t*-tests. For all tests, a *p*-value of < 0.05 was considered significant.

## Results

### SsPepO Binds Plasminogen

**FIGURE 1:**
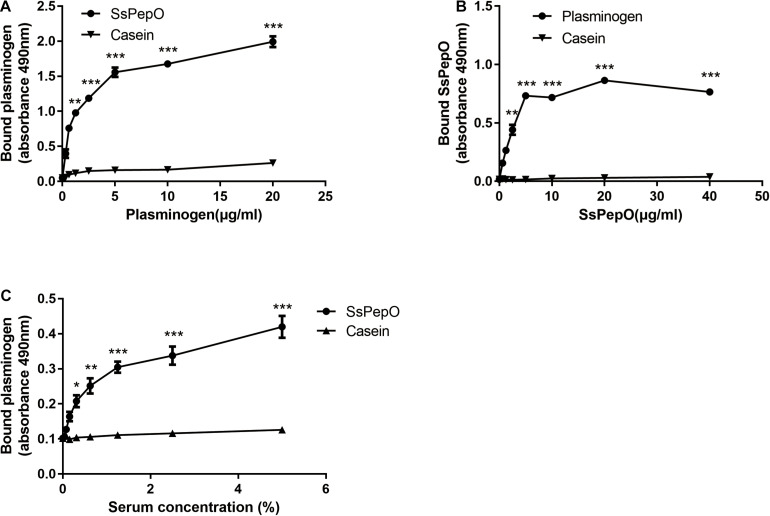
Plasminogen binding to *S. suis* protein endopeptidase O (SsPepO). **(A,B)** Microtiter plates were coated with either SsPepO (5 μg/ml) **(A)** or plasminogen (5 μg/ml) **(B)**, and increasing amounts of plasminogen or SsPepO, respectively, were added. **(C)** The binding of plasminogen to SsPepO from human serum was assessed. Microtiter plates were coated with SsPepO (5 μg/ml), and increasing amounts of serum was added. These assays were repeated three times, and the results were represented as the mean ± SD. ns, not significant; *p < 0.05; **p < 0.01; ***p < 0.001.

To analyze whether SsPepO binds to plasminogen, we performed enzyme-linked immunosorbent assay (ELISA). Microtiter plates coated with SsPepO were incubated with increasing concentrations of plasminogen. As shown in Figure 1A, plasminogen bound immobilized SsPepO in a concentration-dependent manner. Similarly, in a reverse setting, SsPepO also bound immobilized plasminogen in a dose-dependent manner (Figure 1B). Taken together, these data indicate that SsPepO interacts with plasminogen.

To determine if SsPepO could bind plasminogen from human serum, SsPepO was incubated with several dilutions of human serum. As shown in Figure 1C, SsPepO bound to the serum plasminogen in a dose-dependent manner, as determined by an ELISA.

### SsPepO–Plasminogen Binding Depends on Lysine Binding and Ionic Strength

**FIGURE 2:**
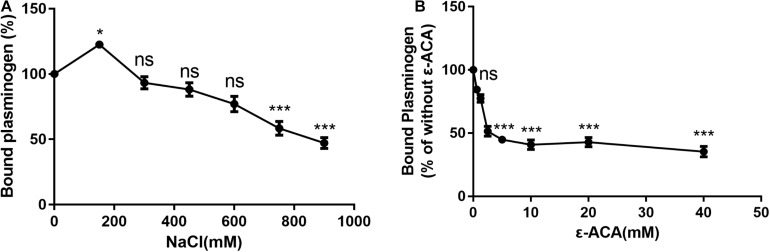
Assessment of factors that may affect SsPepO and plasminogen binding. **(A)** Microtiter plates were coated with SsPepO, and the effect of different concentrations of NaCl on the binding of plasminogen (5 μg/ml) to SsPepO was analyzed. **(B)** The inhibitory effect of the lysine analog ϵ-ACA on the binding of plasminogen to SsPepO was evaluated. These assays were repeated three times, and the results were represented as the mean ± SD. ns, not significant; *p < 0.05; **p < 0.01; ***p < 0.001.

Previous studies have shown that lysine residues and ionic forces play an important role in the interaction of bacterial proteins with plasminogen (Lahteenmaki et al., 2001; Barthel et al., 2012b). Increasing the ionic strength with NaCl significantly decreased the amount of plasminogen binding to SsPepO (Figure 2A). To analyze whether the SsPepO–plasminogen interaction is mediated by lysine residues, SsPepO was incubated with plasminogen in the presence of ϵ-ACA, a lysine analog. The addition of increasing amounts of ϵ-ACA significantly reduced plasminogen binding (Figure 2B).

### Plasminogen and Fibronectin Do Not Compete With One Another for Binding SsPepO

**FIGURE 3:**
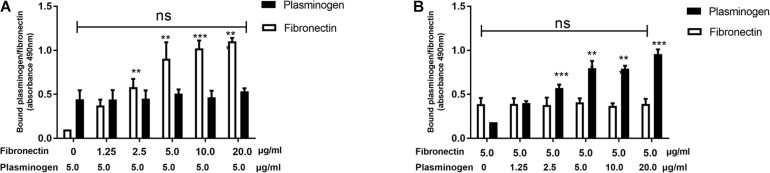
Binding of plasminogen and fibronectin to SsPepO. SsPepO was immobilized on microtiter plates. **(A,B)** A constant amount of plasminogen (5 μg/ml) along with increasing amounts of fibronectin **(A)** or a constant amount of fibronectin (5 μg/ml) along with increasing amounts of plasminogen **(B)** was added. Bound fibronectin and plasminogen were detected using specific Abs. ns, not significant; **p < 0.01; ***p < 0.001.

Our previous study found that SsPepO is a novel fibronectin-binding protein (Liu et al., 2017). To determine whether plasminogen and fibronectin compete with one another for binding SsPepO, a constant concentration of plasminogen and increasing amounts of fibronectin were added to an assay measuring their interaction with SsPepO. Both plasminogen and fibronectin bound to immobilized SsPepO, and the binding of plasminogen was not affected by the increasing amounts of fibronectin (Figure 3A). Similarly, in a reverse setting, the binding of fibronectin was also unaffected by the addition of increasing amounts of plasminogen (Figure 3B). Taken together, the data suggest that plasminogen and fibronectin do not compete with one another for binding SsPepO.

### SsPepO-Bound Plasminogen Degrades the Complement Protein C3b

**FIGURE 4:**
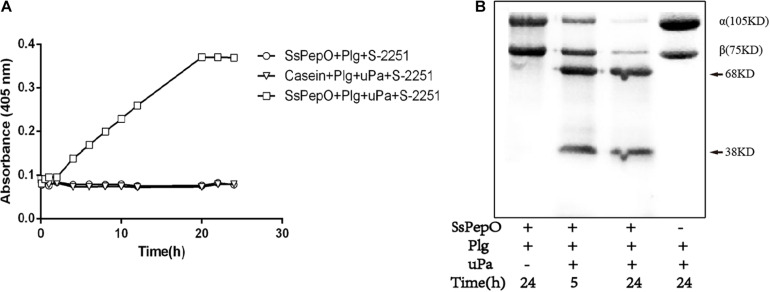
Evaluation of the functionality of SsPepO-bound plasminogen. **(A)** SsPepO (5 μg/well) was immobilized on a microtiter plate. After blocking, these samples were incubated with plasminogen (1 μg/well) in the absence or presence of the urokinase plasmin activator (uPA) together with the chromogenic substrate S-2251. The absorbance at 405 nm was measured to assess conversion of the substrate by the generated plasmin. **(B)** Microtiter plates coated with SsPepO (5 μg/well) were incubated with plasminogen (5 μg/well) followed by addition of uPA (10 units/well) and 125I-C3b, and they were incubated at 37°C. Samples were taken at the indicated timepoints for measurement of C3b degradation. An arrow marks the cleavage products of C3b.

We next tested whether SsPepO-bound plasminogen could be activated by uPA and converted into plasmin *via* proteolytic cleavage. When plasminogen was incubated with SsPepO, followed by the addition of uPA, the plasminogen was converted into plasmin in a time-dependent cleavage manner (Figure 4A). A C3b degradation assay was then performed to analyze whether SsPepO-bound plasminogen can interact with C3b. When C3b was incubated with SsPepO and plasminogen, followed by the addition of uPA, C3b was cleaved by activated plasminogen (plasmin) in a time-dependent cleavage manner (Figure 4B). Following this incubation, the C3b cleavage products were observed as two major bands of 68 and 38 kDa in size. Together, these results show that plasminogen bound to SsPepO is readily converted into the active protease plasmin, which cleaves the complement protein C3b.

### SsPepO Facilitates *S. suis* Binding to Plasminogen

**FIGURE 5:**
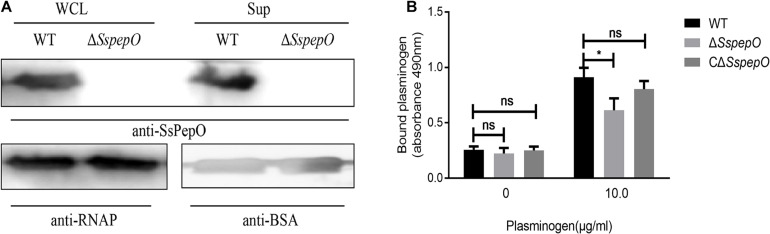
Binding of bacteria to plasminogen. **(A)** Immunoblotting analysis of the SsPepO protein in culture supernatants and whole cell lysates. Bovine serum albumin (BSA) or RNA polymerase (RNAP) was used as the loading control for the supernatant or lysates, respectively. **(B)** Binding of plasminogen to *S. suis*. Microtiter plates were coated with *S. suis* wild-type (WT), SspepO mutant, or CΔ*SspepO* strains, and the binding of plasminogen (0 and 10 μg/ml) was assessed. Bound plasminogen was detected using specific polyclonal Abs. The assay was repeated three times, and the data was represented as the mean ± SD. ns, not significant; *p < 0.05.

The expression levels of SsPepO in WT *S. suis* and Δ*SspepO* were determined in cell surface extracts and culture supernatants by western blot analyses using anti-SsPepO antibody (Figure 5A). The immunoblots confirmed that SsPepO was present in the culture supernatants and bound to the cell surface of WT *S. suis* cells, whereas Δ*SspepO* did not produce SsPepO (Figure 5A). To analyze the role of SsPepO in *S. suis* binding to plasminogen, we performed a direct binding assay using these three types of bacteria. Compared with WT *S. suis*, the Δ*SspepO* mutant showed a significantly lower plasminogen-binding ability and the CΔ*SspepO* restored the ability (Figure 5B). Therefore, the SsPepO secreted by *S. suis* in culture supernatant or bound to the cell surface likely contributes to plasminogen binding.

### SsPepO Mediates *S. suis* Adherence to and Invasion of RBMECs

**FIGURE 6:**
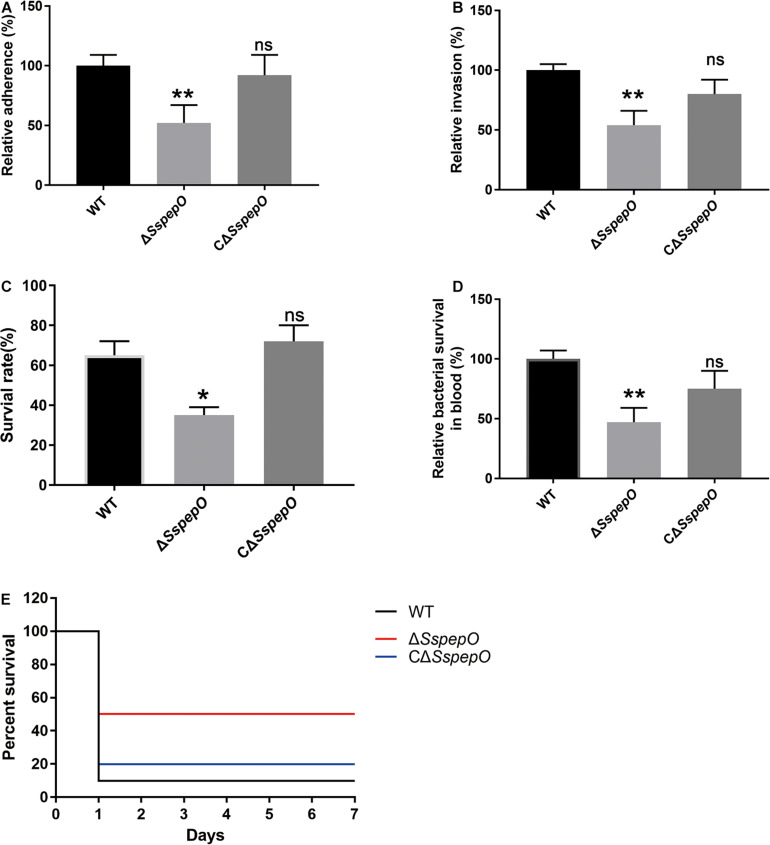
Effect of *S. suis* SsPepO on adherence to and invasion of rat brain microvessel endothelial cell (RBMECs) and virulence. **(A,B)** Adherence to **(A)** and invasion of **(B)** WT *S. suis*, Δ*SspepO*, and CΔ*SspepO* were determined by counting the colony-forming units (CFU) per well obtained from sample aliquots plated onto blood agar plates after 2 h of infection. **(C,D)** WT *S. suis*, Δ*SspepO*, and CΔ*SspepO* strains were added to serum **(C)** or blood **(D)**, and their survival rates were calculated. These assays were repeated three times, and the results were represented as the mean ± SD. **(E)** Each *S. suis* strain (8 × 10^8^ CFU) was injected into mice, and the survival of the mice was monitored for 7 days. *p < 0.05.

To better elucidate the role of SsPepO in *S. suis* adherence to and invasion of host cells, the adhesion and invasion abilities of WT *S. suis*, Δ*SspepO*, and CΔ*SspepO* were compared. RBMECs were infected with WT *S. suis*, Δ*SspepO*, or CΔ*SspepO* for 2 h. Compared with WT *S. suis*, the Δ*SspepO* mutant showed a significant reduction in both adherence to and invasion of RBMECs and the CΔ*SspepO* restored these abilities (Figures 6A,B). The results of these experiments suggest that SsPepO facilitates *S. suis* attachment to and internalization into host cells.

### SsPepO Facilitates Survival of *S. suis* in Serum and Blood and Modulates *S. suis* Virulence

To evaluate the impact of SsPepO deletion during systemic infections, we performed serum survival assay and whole blood bactericidal assay. Compared with WT *S. suis*, the Δ*SspepO* mutant showed a significant reduction in resistance to serum and whole blood and the CΔ*SspepO* restored the ability (Figures 6C,D). To analyze the virulence of *S. suis* strains infection *in vivo*, we employed a murine model (Liu et al., 2017). All the WT *S. suis*-infected mice developed typical clinical symptoms of *S. suis* infection, including a rough hair coat, shivering, and lethargy. In contrast, all the Δ*SspepO* mutant-infected mice showed mild or no clinical signs of infection. The survival ratio of the WT *S. suis*-infected mice and the CΔ*SspepO*-infected mice was lower than that of the Δ*SspepO* mutant-infected mice (Figure 6E). The results indicate that SsPepO plays an important role in *S. suis* infection.

## Discussion

The pathogenesis of *S. suis* meningitis involves the hematogenous spread and entry into the central nervous system (CNS) of the bacteria *via* invading the blood–brain barrier (van Samkar et al., 2015). However, the precise mechanism whereby *S. suis* leaves the bloodstream and gains access to the CNS is still being elucidated. Our previous studies showed that SsPepO is a fibronectin-binding protein that facilitates the development of meningitis in *S. suis* (Tan et al., 2011). Here, we further demonstrate that SsPepO is a plasminogen-binding protein that plays an important role in the immune evasion of *S. suis.*

Previous studies showed that various bacterial pathogens have developed numerous ways to interact with plasminogen and use the host’s powerful proteolytic system to their own advantage (Peetermans et al., 2016). A previous study reported that PepO promotes macrophage phagocytosis and bactericidal activity (Shu et al., 2020). *Streptococcus pneumoniae* PepO facilitates bacterial colonization and internalization (Agarwal et al., 2013). *Streptococcus pyogenes* PepO is involved in complement evasion by binding to C1q (Honda-Ogawa et al., 2017). Plasmin blocks complement cascade by degrading C3b and C5 (Barthel et al., 2012a). In this study, we demonstrated that SsPepO binds plasminogen in a dose-dependent manner and that the SsPepO–plasminogen interaction is affected by ionic strength as well as the lysine analog ϵ-ACA. This type of interaction with plasminogen is similar to that observed for pneumococcal PepO, pneumococcal enolase, and *Haemophilus influenzae* protein E (Bergmann et al., 2005; Hallstrom et al., 2010; Barthel et al., 2012b; Agarwal et al., 2013). In addition, we demonstrated that SsPepO could bind to plasminogen from human serum and the plasminogen was readily activated by uPA to plasmin that subsequently cleaved C3b. Pneumococcal PepO and *H. influenzae* protein E can also cleave C3b *via* a similar mechanism (Segura, 2009; Barthel et al., 2012b). Our results showed that WT *S. suis* and CΔ*SspepO* were more resistant to serum and whole blood than the Δ*SspepO*. The result was in accordance with a previous report that showed that pepO mutants diminished in blood (Alves et al., 2020). The result might be due to plasminogen bound to SsPepO is readily converted into the active protease plasmin, which cleaves the complement protein C3b and thus contributes to evasion from complement mediated killing.

Fibronectin is an ECM protein involved in several biological processes and is also recognized as a receptor by many bacterial pathogens (Hymes and Klaenhammer, 2016). Ssa and FBPS are fibronectin-binding protein. Previous studies reported that Ssa and FBPS contribute to pathogenesis of *S. suis* (de Greeff et al., 2002; Li et al., 2013). *Staphylococcus aureus* FnBPB promotes plasminogen conversion to plasmin (Pietrocola et al., 2019). *Streptococcus pneumoniae* PavA and pfbA are essential for virulence (Holmes et al., 2001; Yamaguchi et al., 2008). Here, we have shown that SsPepO binds fibronectin, in addition to binding plasminogen. Furthermore, our results demonstrate that plasminogen and fibronectin do not compete with one another for binding SsPepO. Many other bacterial proteins can also bind to more than one ECM protein; for example, S*taphylococcus aureus* FnBPB is a fibrinogen-binding protein as well as a fibronectin-binding protein and *Mycoplasma synoviae* enolase is a plasminogen-binding protein as well as a fibronectin-binding protein (Bao et al., 2014; Pietrocola et al., 2016).

The *SspepO*-deficient mutant showed a significant reduction in binding to plasminogen compared with the WT *S. suis*. Additionally, our data also indicate that SsPepO facilitates *S. suis* adhesion to and invasion of host cells. These *in vitro* results were corroborated by our *in vivo* findings in a murine model of *S. suis* hematogenous meningitis.

Infection with the *SspepO*-deficient mutant resulted in a lower mortality rate compared with the WT *S. suis* and CΔ*SspepO*. Our findings confirm that SsPepO plays an important role in the virulence of *S. suis*, in agreement with the previously published data from a porcine model (Tan et al., 2011).

**FIGURE 7:**
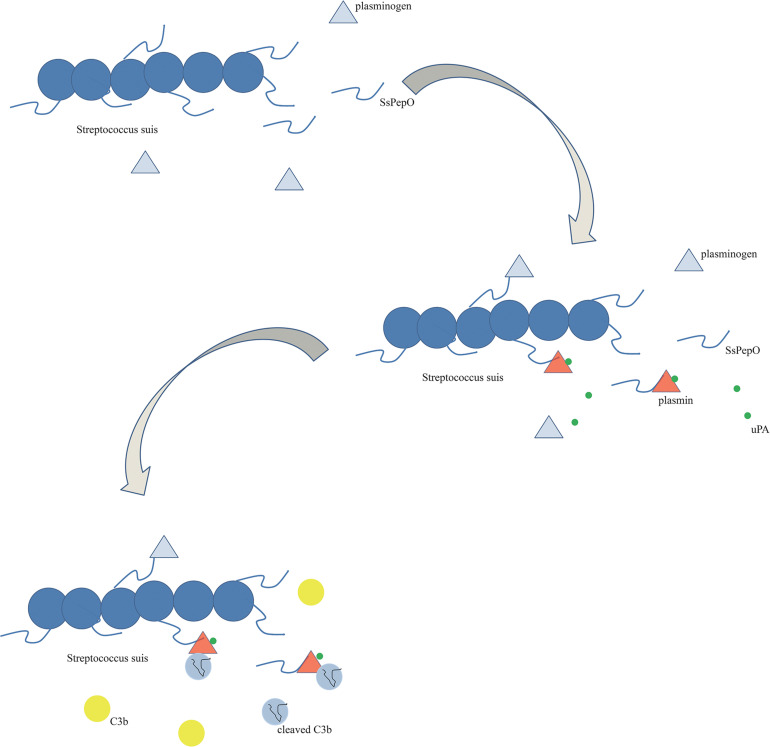
Schematic diagram. SsPepO binds plasminogen. The binding of SsPepO and plasminogen, upon the activation of urokinase-type plasminogen activator, generated plasmin, which could cleave complement C3b.

In conclusion, the results of this study suggest that SsPepO is a newly identified virulence factor facilitating the survival of *S. suis* in whole blood. SsPepO, as a plasminogen- and fibronectin-binding protein, facilitates *S. suis* adherence to and internalization into host epithelial and endothelial cells. Besides having plasminogen-binding activity, SsPepO can also cleave C3b *via* activated plasmin, which is a method of mediating complement control (Figure 7).

## Data Availability Statement

The original contributions presented in the study are included in the article/supplementary material, further inquiries can be directed to the corresponding author/s.

## Ethics Statement

The animal study was reviewed and approved by Scientific Ethics Committee of Huazhong Agricultural University.

## Author Contributions

WB, FY, KY, JL, YZ, and HC conceived and designed the experiments. All authors contributed to the acquisition and analysis of the data and the writing of the manuscript.

## Conflict of Interest

The authors declare that the research was conducted in the absence of any commercial or financial relationships that could be construed as a potential conflict of interest.

## References

[B1] Agarwal V. Kuchipudi A. Fulde M. Riesbeck K. Bergmann S. Blom A. M. (2013). Streptococcus pneumoniae endopeptidase O (PepO) is a multifunctional plasminogen- and fibronectin-binding protein, facilitating evasion of innate immunity and invasion of host cells. *J. Biol. Chem.* 288 6849–6863. 10.1074/jbc.m112.405530 23341464 PMC3591595

[B2] Alves L. A. Ganguly T. Harth-Chu E. N. Kajfasz J. Lemos J. A. Abranches J. (2020). PepO is a target of the two-component systems VicRK and CovR required for systemic virulence of Streptococcus mutans. *Virulence* 11 521–536. 10.1080/21505594.2020.1767377 32427040 PMC7239026

[B3] Bao S. Guo X. Yu S. Ding J. Tan L. Zhang F. (2014). Mycoplasma synoviae enolase is a plasminogen/fibronectin binding protein. *BMC Vet. Res.* 10:223. 10.1186/s12917-014-0223-6 25253294 PMC4189797

[B4] Barthel D. Schindler S. Zipfel P. F. (2012a). Plasminogen is a complement inhibitor. *J. Biol. Chem.* 287 18831–18842. 10.1074/jbc.m111.323287 22451663 PMC3365705

[B5] Barthel D. Singh B. Riesbeck K. Zipfel P. F. (2012b). *Haemophilus influenzae* uses the surface protein E to acquire human plasminogen and to evade innate immunity. *J. Immunol.* 188 379–385. 10.4049/jimmunol.1101927 22124123

[B6] Bergmann S. Rohde M. Preissner K. T. Hammerschmidt S. (2005). The nine residue plasminogen-binding motif of the pneumococcal enolase is the major cofactor of plasmin-mediated degradation of extracellular matrix, dissolution of fibrin and transmigration. *Thromb. Haemost.* 94 304–311. 16113819 10.1160/TH05-05-0369

[B7] Brassard J. Gottschalk M. Quessy S. (2004). Cloning and purification of the Streptococcus suis serotype 2 glyceraldehyde-3-phosphate dehydrogenase and its involvement as an adhesin. *Vet. Microbiol.* 102 87–94. 10.1016/j.vetmic.2004.05.008 15288930

[B8] Castiblanco-Valencia M. M. Fraga T. R. Pagotto A. H. Serrano S. M. Abreu P. A. Barbosa A. S. (2016). Plasmin cleaves fibrinogen and the human complement proteins C3b and C5 in the presence of Leptospira interrogans proteins: a new role of LigA and LigB in invasion and complement immune evasion. *Immunobiology* 221 679–689. 10.1016/j.imbio.2016.01.001 26822552

[B9] Chen C. Tang J. Dong W. Wang C. Feng Y. Wang J. (2007). A glimpse of streptococcal toxic shock syndrome from comparative genomics of S. suis 2 Chinese isolates. *PLoS One* 2:e315. 10.1371/journal.pone.0000315 17375201 PMC1820848

[B10] de Greeff A. Buys H. Verhaar R. Dijkstra J. van Alphen L. Smith H. E. (2002). Contribution of fibronectin-binding protein to pathogenesis of Streptococcus suis serotype 2. *Infect. Immun.* 70 1319–1325. 10.1128/iai.70.3.1319-1325.2002 11854216 PMC127759

[B11] Dong X. Chao Y. Zhou Y. Zhou R. Zhang W. Fischetti V. A. (2021). The global emergence of a novel *Streptococcus suis* clade associated with human infections. *EMBO Mol. Med.* e13810. 10.15252/emmm.202013810 34137500 PMC8261479

[B12] Esgleas M. Li Y. Hancock M. A. Harel J. Dubreuil J. D. Gottschalk M. (2008). Isolation and characterization of alpha-enolase, a novel fibronectin-binding protein from Streptococcus suis. *Microbiology* 154 2668–2679. 10.1099/mic.0.2008/017145-0 18757800

[B13] Feng Y. Zhang H. Ma Y. Gao G. F. (2010). Uncovering newly emerging variants of Streptococcus suis, an important zoonotic agent. *Trends Microbiol.* 18 124–131. 10.1016/j.tim.2009.12.003 20071175

[B14] Gottschalk M. Segura M. (2000). The pathogenesis of the meningitis caused by Streptococcus suis: the unresolved questions. *Vet. Microbiol.* 76 259–272. 10.1016/s0378-1135(00)00250-9 10973700

[B15] Gottschalk M. Xu J. Calzas C. Segura M. (2010). Streptococcus suis: a new emerging or an old neglected zoonotic pathogen? *Future Microbiol.* 5 371–391. 10.2217/fmb.10.2 20210549

[B16] Hallstrom T. Haupt K. Kraiczy P. Hortschansky P. Wallich R. Skerka C. (2010). Complement regulator-acquiring surface protein 1 of Borrelia burgdorferi binds to human bone morphogenic protein 2, several extracellular matrix proteins, and plasminogen. *J. Infect. Dis.* 202 490–498. 10.1086/653825 20565259

[B17] Hatrongjit R. Kerdsin A. Gottschalk M. Takeuchi D. Hamada S. Oishi K. (2015). First human case report of sepsis due to infection with Streptococcus suis serotype 31 in Thailand. *BMC Infect. Dis.* 15:392. 10.1186/s12879-015-1136-0 26420029 PMC4588491

[B18] Hoa N. T. Chieu T. T. Do Dung S. Long N. T. Hieu T. Q. Luc N. T. (2013). Streptococcus suis and porcine reproductive and respiratory syndrome. *Vietnam Emer Infec Dis* 19 331–333. 10.3201/eid1902.120470 PMC3559037 23343623

[B19] Holmes A. R. McNab R. Millsap K. W. Rohde M. Hammerschmidt S. Mawdsley J. L. (2001). The pavA gene of Streptococcus pneumoniae encodes a fibronectin-binding protein that is essential for virulence. *Mol. Microbiol.* 41 1395–1408. 10.1046/j.1365-2958.2001.02610.x 11580843

[B20] Honda-Ogawa M. Sumitomo T. Mori Y. Hamd D. T. Ogawa T. Yamaguchi M. (2017). Streptococcus pyogenes Endopeptidase O Contributes to Evasion from Complement-mediated Bacteriolysis via Binding to Human Complement Factor C1q. *Biol. Chem.* 292 4244–4254. 10.1074/jbc.m116.749275 28154192 PMC5354489

[B21] Hymes J. P. Klaenhammer T. R. (2016). Stuck in the middle: fibronectin-binding proteins in gram-positive bacteria. *Front. Microbiol.* 7:1504. 10.3389/fmicb.2016.01504 27713740 PMC5031765

[B22] Jobin M. C. Brassard J. Quessy S. Gottschalk M. Grenier D. (2004). Acquisition of host plasmin activity by the Swine pathogen Streptococcus suis serotype 2. *Infect. Immun.* 72 606–610. 10.1128/iai.72.1.606-610.2004 14688145 PMC343993

[B23] Lahteenmaki K. Kuusela P. Korhonen T. K. (2001). Bacterial plasminogen activators and receptors. *FEMS Microbiol. Rev.* 25 531–552. 10.1016/s0168-6445(01)00067-5 11742690

[B24] Li W. Wan Y. Tao Z. Chen H. Zhou R. (2013). A novel fibronectin-binding protein of Streptococcus suis serotype 2 contributes to epithelial cell invasion and in vivo dissemination. *Vet. Microbiol.* 162 186–194. 10.1016/j.vetmic.2012.09.004 23021642

[B25] Liu F. Li J. Yan K. Li H. Sun C. Zhang S. (2017). Binding of Fibronectin to SsPepO Facilitates the Development of Streptococcus suis Meningitis. *J. Infect. Dis.* 217 973–982. 10.1093/infdis/jix523 29253192

[B26] Liu P. Pian Y. Li X. Liu R. Xie W. Zhang C. (2014). Streptococcus suis adenosine synthase functions as an effector in evasion of PMN-mediated innate immunit. *J. Infect. Dis.* 210 35–45. 10.1093/infdis/jiu050 24446521

[B27] Musyoki A. M. Shi Z. Xuan C. Lu G. Qi J. Gao F. (2016). Structural and functional analysis of an anchorless fibronectin-binding protein FBPS from Gram-positive bacterium Streptococcus suis. *Proc. Natl. Acad. Sci. U.S.A.* 113 13869–13874. 10.1073/pnas.1608406113 27834729 PMC5137682

[B28] Normile D. (2005). Infectious diseases. WHO probes deadliness of China’s pig-borne disease. *Science* 309 1308–1309. 10.1126/science.309.5739.1308a 16123268

[B29] Peetermans M. Vanassche T. Liesenborghs L. Lijnen R. H. Verhamme P. (2016). Bacterial pathogens activate plasminogen to breach tissue barriers and escape from innate immunity. *Crit. Rev. Microbiol.* 42 866–882. 10.3109/1040841x.2015.1080214 26485450

[B30] Pian Y. Li X. Zheng Y. Wu X. Yuan Y. Jiang Y. (2016). Binding of Human Fibrinogen to MRP Enhances Streptococcus suis Survival in Host Blood in a alphaXbeta2 Integrin-dependent Manner. *Sci. Rep.* 6:26966. 27231021 10.1038/srep26966 PMC4882601

[B31] Pietrocola G. Nobile G. Gianotti V. Zapotoczna M. Foster T. J. Geoghegan J. A. (2016). Molecular Interactions of Human Plasminogen with Fibronectin-binding Protein B (FnBPB), a Fibrinogen/Fibronectin-binding Protein from Staphylococcus aureus. *J. Biol. Chem.* 291 18148–18162. 10.1074/jbc.m116.731125 27387503 PMC5000064

[B32] Pietrocola G. Nobile G. Alfeo M. J. Foster T. J. Geoghegan J. A. De Filippis V. (2019). Fibronectin-binding protein B (FnBPB) from Staphylococcus aureus protects against the antimicrobial activity of histones. *J. Biol. Chem.* 294 3588–3602. 10.1074/jbc.ra118.005707 30622139 PMC6416437

[B33] Segura M. (2009). Streptococcus suis: an emerging human threat. *J. Infect Dis.* 199 4–6. 10.1086/594371 19016626

[B34] Shu Z. Yuan J. Wang H. Zhang J. Li S. Zhang H. (2020). Streptococcus pneumoniae PepO promotes host anti-infection defense via autophagy in a Toll-like receptor 2/4 dependent manner. *Virulence* 11 270–282. 10.1080/21505594.2020.1739411 32172666 PMC7161686

[B35] Singh B. Fleury C. Jalalvand F. Riesbeck K. (2012). Human pathogens utilize host extracellular matrix proteins laminin and collagen for adhesion and invasion of the host. *FEMS Microbiol. Rev.* 36 1122–1180. 10.1111/j.1574-6976.2012.00340.x 22537156

[B36] Singh B. Al-Jubair T. Morgelin M. Thunnissen M. M. Riesbeck K. (2013). The unique structure of *Haemophilus influenzae* protein E reveals multiple binding sites for host factors. *Infect. Immun.* 81 801–814. 10.1128/iai.01111-12 23275089 PMC3584867

[B37] Singh B. Al-Jubair T. Voraganti C. Andersson T. Mukherjee O. Su Y. C. (2015). *Moraxella* catarrhalis Binds Plasminogen To Evade Host Innate Immunity. *Infect. Immun.* 83 3458–3469. 10.1128/iai.00310-15 26099590 PMC4534650

[B38] Sun Y. Li N. Zhang J. Liu H. Liu J. Xia X. (2016). enolase of streptococcus suis serotype 2 enhances blood-brain barrier permeability by inducing il-8 release. *Inflammation* 39 718–726. 10.1007/s10753-015-0298-7 26732390

[B39] Tan C. Liu M. Li J. Jin M. Bei W. Chen H. (2011). SsPep contributes to the virulence of Streptococcus suis. *Microb. Pathog.* 51 319–324. 10.1016/j.micpath.2011.07.008 21839825

[B40] van Samkar A. Brouwer M. C. Schultsz C. van der Ende A. van de Beek D. (2015). Streptococcus suis meningitis: a systematic review and meta-analysis. *PLoS Negl. Trop. Dis.* 9:e0004191. 10.1371/journal.pntd.0004191 26505485 PMC4624688

[B41] Wang J. Kong D. Zhang S. Jiang H. Zheng Y. Zang Y. (2015). Interaction of fibrinogen and muramidase-released protein promotes the development of Streptococcus suis meningitis. *Front. Microbiol.* 6:1001. 10.3389/fmicb.2015.01001 26441928 PMC4585153

[B42] Wertheim H. F. Nghia H. D. Taylor W. Schultsz C. (2009). Streptococcus suis: an emerging human pathogen. *Clin. Infect. Dis.* 48 617–625. 19191650 10.1086/596763

[B43] Westerlund B. Korhonen T. K. (1993). Bacterial proteins binding to the mammalian extracellular matrix. *Mol. Microbiol.* 9 687–694. 10.1111/j.1365-2958.1993.tb01729.x 7901732

[B44] Wisselink H. J. Smith H. E. Stockhofe-Zurwieden N. Peperkamp K. Vecht U. (2000). Distribution of capsular types and production of muramidase-released protein (MRP) and extracellular factor (EF) of Streptococcus suis strains isolated from diseased pigs in seven European countries. *Vet. Microbiol.* 74 237–248. 10.1016/s0378-1135(00)00188-7 10808092

[B45] Yamaguchi M. Terao Y. Mori Y. Hamada S. Kawabata S. (2008). PfbA, a novel plasmin- and fibronectin-binding protein of Streptococcus pneumoniae, contributes to fibronectin-dependent adhesion and antiphagocytosis. *J. Biol. Chem* 283 36272–36279. 10.1074/jbc.m807087200 18974092 PMC2662297

[B46] Yu H. Jing H. Chen Z. Zheng H. Zhu X. Wang H. (2006). Human Streptococcus suis outbreak, Sichuan, China. *Emerg. Infect. Dis.* 12 914–920. 16707046 10.3201/eid1206.051194 PMC3373052

